# Ultrasound-assisted lipase-catalyzed synthesis of D-isoascorbyl palmitate: process optimization and Kinetic evaluation

**DOI:** 10.1186/1752-153X-7-180

**Published:** 2013-12-09

**Authors:** Feng-Jie Cui, Hong-Xia Zhao, Wen-Jing Sun, Zhuan Wei, Si-Lian Yu, Qiang Zhou, Ying Dong

**Affiliations:** 1 School of Food and Biological Engineering, Jiangsu University, Zhenjiang 212013, P.R. China; 2 Parchn Sodium Isovitamin C Co. Ltd, Dexing 334221, P.R. China; 3 Jiangxi Provincial Engineering and Technology Center for Food Additives Bio-production, Xingangshan Town, Dexing 334221, P.R. China; 4 Department of Pharmaceutical, Hebei Chemical and Pharmaceutical College, Shijiazhuang 050026, P.R. China

**Keywords:** D-isoascorbyl palmitate, Ultrasound treatment, Process optimization, Kinetic evaluation

## Abstract

**Background:**

D-isoascorbic acid is a food antioxidant additive and used in accordance with Good Manufacturing Practice (GMP). High solubility in water (about 150 g/L at 25°C) reduces its effectiveness in stabilizing fats and oils. Our research group had successfully synthesized D-isoascorbyl palmitate using immobilized lipase Novozym 435 as a biocatalyst. Low production efficiency of D-isoascorbyl palmitate is still a problem for industrial production due to the long reaction time of over 24 h. In the present work, ultrasonic treatment was applied for accelerating the reaction process. The operation parameters were optimized to obtain the maximum D-isoascorbyl palmitate conversion rate by using a 5-level-4-factor Central Composite Design (CCD) and Response Surface Methdology (RSM). The reaction apparent kinetic parameters under the ultrasound treatment and mechanical shaking conditions were also determined and compared.

**Results:**

Results showed that ultrasound treatment decreased the reaction time by over 50%. D-isoascorbyl palmitate yielded to 94.32 ± 0.17% and the productivity reached to 8.67 g L^-1^ h^-1^ under the optimized conditions as: 9% of enzyme load (w/w), 61°C of reaction temperature, 1:5 of D- isoascorbic-to-palmitic acid molar ratio, and 137 W of the ultrasound power. The immobilized lipase Novozym 435 could be reused for 7 times with 65% of the remained D-isoascorbyl palmitate conversion rate. The reaction kinetics showed that the maximum apparent reaction rate (*v*_max_) of the ultrasound-assisted reaction was 2.85 times higher than that of the mechanical shaking, which proved that ultrasound treatment significantly enhanced the reaction efficiency.

**Conclusion:**

A systematic study on ultrasound-assisted enzymatic esterification for D-isoascorbyl palmitate production is reported. The results show a promising perspective of the ultrasound technique to reduce the reaction time and improve the production efficiency. The commercial D-isoascorbyl palmitate synthesis will be potentially realized due to this ultrasound-promoted esters synthesis method.

## Background

Lipid oxidation is the major cause for development of rancidity and off-flavor compounds in edible fatty products such as peroxides, ketones, aldehydes and oxyacids [1,2]. It will decrease the nutritional values of fats, oils and food materials and their shelf life. Therefore, to screen and/or develop the favorable additives for increasing the shelf of edible fatty products and slowing the oxidative processes are the main challenges for the oil-based food industries [3].

D- isoascorbic acid (synonyms: Erythorbic acid, D-araboascorbic acid) is mainly used as a food antioxidant with the ability to prevent the food oxidation, decrease the color aroma and flavors, and block the carcinogen ammonium nitrite production during food manufacturing process [4]. Food and Drug Administration (FDA) had classified it as generally recognized as safe (GRAS) additives [5]. Now it is added in processed foods in accordance with Good Manufacturing Practice (GMP) [6,7].

D-isoascorbic acid is freely soluble in water (150 g/L at 25°C). However, the high hydrophilicity prevents its application in some fats, oil-based foods or cosmetics [8,9]. Converting D-isoascorbic acid into oil-soluble fatty acid ester is an effective solution to alter its solubility for enlarging its applications to the oil food, cosmetics and pharmaceutical fields. Recently, an erythorbyl fatty acid ester of erythorbyl laurate was obtained for improving the lipophilicity [10,11]. Our research group had also successfully synthesized D-isoascorbyl palmitate using immobilized lipase Novozym 435 as a biocatalyst and optimized the process parameters for obtaining the maximum D-isoascorbyl palmitate yield [12]. However, the long reaction time over 24 h is still a drawback for industrial production and results in the low conversion efficiency. Hence, decreasing the reaction time and increasing the conversional efficiency are still a valuable consideration for D-isoascorbyl fatty acid ester synthesis.

Ultrasound has recently been applied for synthesizing the target compounds since it can increase the reaction process and improve the efficiency under the mild reaction conditions [13-17]. For example, the enzymatic-catalyzed transesterification process of glucose and divinyl dicarboxylates was significantly accelerated by using ultrasound assisted treatment [18]. Ultrasonication increased the biodiesel conversion to 85.5% from non-edible vegetable oil with the immobilized lipase (*Chromobacterium viscosum*) as a catalyst [19] and also decreased the reaction time of ascorbyl palmitate to 2 h with the conversion of about 27% [20]. Ultrasound-induced cavitation bubbles collapse [14,15] and efficient stirring or mixing of the layers might contribute to the increase of the chemical and/or enzymatic reactions rates in heterogeneous reactions [16,17]. However, few references are available for applying the ultrasound treatment in the isoascorbyl esters synthesis process.

In the present study, lipase-catalyzed synthesis of D-isoascorbyl palmitate under the ultrasound treatment using immobilized lipase Novozym 435 as a biocatalyst was investigated. 5-level-4-factor Central Composite Design (CCD) and response surface methodology (RSM) were applied to find the relationships between reaction parameters and the D-isoascorbyl palmitate conversion rate and maximizing the D-isoascorbyl palmitate production efficiency. The process kinetics was finally developed for comparison of the ultrasound and mechanical shaking treatments.

## Results and discussion

### Optimization of the conversion rate of D-isoascorbyl palmitate under the ultrasound treatment

**Figure 1:**
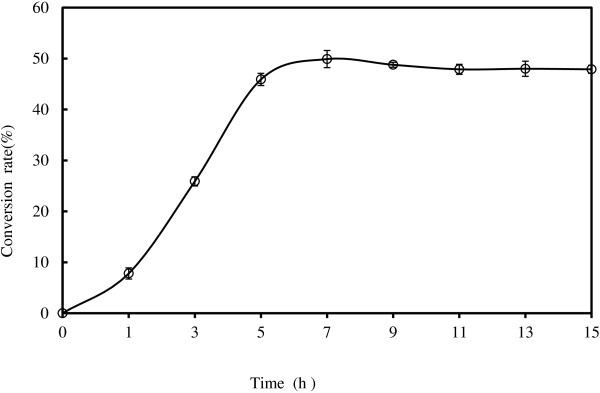
**Time course of lipase catalyzed synthesis of D-isoascorbyl palmitate under ultrasound-assisted treatment.** (Enzyme load 10% (weight % of substrates); temperature: 50°C; molar ratio: 1:4; acetone 20 mL; 4 Å molecular sieves content: 50 g/L; Power: 180 W).

Firstly, the time course of lipase-catalyzed synthesis D- isoascorbyl palmitate from D-isoascorbic and palmitic acid with ultrasound treatment was obtained to select the optimal reaction time for the next statistical experiments. As shown in Figure 1, the conversion rate increased rapidly to stable level of 48.68% during the 6-h reaction when the reaction condition was set as following:enzyme load of 10% (w/w), reaction temperature of 50°C and D- isoascorbic-to-palmitic acid molar ratio of 1:4, acetone 20 mL, 50 g/L of molecular sieves content and 180 W ultrasound power. Hence, 6-h of reaction time was selected for the remaining tests.

Response surface methodology is an empirical modeling technique used to evaluate the relationship between a set of controllable experimental factors and the observed results. In order to systemically find the relationships between reaction temperature, substrate molar ratio, enzyme load, and ultrasonic power for the synthesis of D-isoascorbyl palmitate, a 5-level-4-factor Central Composite Design (CCD) was applied with the 30 total experiments. Table 1 presented the experimental design and results of ultrasound assisted D-isoascorbyl palmitate synthesis using Novozym 435 as a biocatalyst within the 6-h reaction. From Table 1, the run #1 and #16 had the minimum and maximum D-isoascorbyl palmitate conversion rates of 38.25% and 91.89%, respectively. Other experimental runs presented the conversion rate of over 50%.

**Table 1:** Experimental designs and the results of CCD

**Run**	**A(X1)**^**a**^	**B( *X *** **2)**^**b**^	**C(X3)**^**c**^	**D(X4)**^**d**^	**Conversion rate (%)**			
					**I II Average predicted**			
1	-1	-1	-1	-1	49.00	37.50	38.25 ± 1.06	40.81
2	1	-1	-1	-1	49.77	47.47	48.62 ± 1.63	49.85
3	-1	1	-1	-1	70.77	67.95	69.36 ± 2.00	69.55
4	1	1	-1	-1	72.66	69.04	70.85 ± 2.56	73.65
5	-1	-1	1	-1	45.99	34.31	40.15 ± 8.26	46.72
6	1	-1	1	-1	51.90	56.00	53.95 ± 2.90	57.31
7	-1	1	1	-1	77.98	77.50	77.74 ± 0.34	75.84
8	1	1	1	-1	81.98	79.62	80.8 ± 1.68	81.80
9	-1	-1	-1	1	60.21	56.53	58.37 ± 2.60	61.21
10	1	-1	-1	1	66.99	71.85	69.42 ± 3.44	73.48
11	-1	1	-1	1	76.09	78.47	77.28 ± 1.69	76.08
12	1	1	-1	1	85.99	86.91	86.45 ± 0.65	83.42
13	-1	-1	1	1	65.33	70.65	67.99 ± 3.76	67.34
14	1	-1	1	1	76.00	79.62	77.81 ± 2.55	81.86
15	-1	1	1	1	82.98	77.58	80.28 ± 3.83	82.59
16	1	1	1	1	90.09	93.69	91.89 ± 2.55	91.48
17	-2	0	0	0	62.90	56.84	59.87 ± 4.29	57.35
18	2	0	0	0	78.90	78.02	78.46 ± 0.63	75.27
19	0	-2	0	0	56.09	63.65	59.87 ± 5.34	51.05
20	0	2	0	0	88.98	85.02	87.00 ± 2.80	90.11
21	0	0	-2	0	70.02	60.50	65.26 ± 6.73	63.39
22	0	0	2	0	80.99	81.39	81.19 ± 0.28	77.36
23	0	0	0	-2	63.60	57.30	60.45 ± 4.45	55.54
24	0	0	0	2	85.99	87.43	86.71 ± 1.02	85.07
25	0	0	0	0	56.99	59.93	58.46 ± 2.07	58.43
26	0	0	0	0	58.46	59.45	58.95 ± 0.07	58.43
27	0	0	0	0	60.09	56.83	58.46 ± 2.31	58.43
28	0	0	0	0	58.46	57.51	57.98 ± 0.67	58.43
29	0	0	0	0	60.82	55.18	58.00 ± 3.99	58.43
30	0	0	0	0	58.46	59.01	58.73 ± 0.39	58.43

a: X1:Temperature; b: *X*2: Molar ratio (D-isoascorbic: palmitic acid);

c: X3: Enzyme load; d: X4: Ultrasound power.

Table 2 summarized the analysis of variance (ANOVA) for checking accuracy of the polynomial model. The model well presented the relationship between the responses and the variables with the model *F*-value of 20.67 and low *p-*value (*p <* 0.0001). Values of “Probability > *F*” less than 0.05 indicate the model terms are significant. In general, higher *F*-value means the more significance of the corresponding coefficient [21]. From Table 2, the most influencing variables on the model response were *X*_2_ (Molar ratio of D-isoascorbic to palmitic acid) and X_4_ (ultrasonic power).

**Table 2:** ANOVA for response surface quadratic model

**Source**	**Sum of squares**	**df**	**Mean square**	**F -value**	**p-value**
Model	5292.98	14	378.07	20.67	< 0.0001**
A	481.91	1	481.91	26.35	0.0001**
B	2288.43	1	2288.43	125.12	< 0.0001**
C	292.92	1	292.92	16.02	0.0012**
D	1384.64	1	1384.64	75.71	< 0.0001**
AB	24.27	1	24.27	1.33	0.2674
AC	2.41	1	2.41	0.13	0.7219
AD	10.44	1	10.44	0.57	0.4616
BC	0.15	1	0.15	0.01	0.9301
BD	192.20	1	192.20	10.51	0.0055**
CD	0.05	1	0.05	0.00	0.9599
A^2^	106.51	1	106.51	5.82	0.0291*
B^2^	253.27	1	253.27	13.85	0.0020**
C^2^	244.59	1	244.59	13.37	0.0023**
D^2^	259.45	1	259.45	14.19	0.0019**
Residual	274.34	15	18.29		
Lack of fit	273.59	10	27.36	182.63	< 0.0001
Pure error	0.75	5	0.15		
Cor total	5567.33	29			
R-squared = 0.9570		Adj-squared = 0.9047		Ade precision = 16.756	

**Significant at 1% level *Significant at 5% level C.V. % = 6.36.

The determination coefficient (*R*^2^) and adjusted determination coefficient (Adj. *R*^*2*^) are commonly used to check the goodness of model. In the present study, *R*^*2*^ of 0.9570 implied that 95.7% of the variation in the production yield could be explained by the regression model. The relatively low coefficient of variation value (C.V. % =6.36) also proved the remarkable precision and reliability of the model. Neglecting the statistically insignificant terms (*P* > 0.05) and recalculating the coefficients, the quadratic models for D-isoascorbyl palmitate conversion ratio in terms of coded factors are presented as follows:

(1) $$\begin{array}{c} Y=58.43+4.48X_{1}+9.76X_{2}+3.49X_{3}\\ \;+7.60X_{4}-3.47X_{2}X_{4}+1.97{X_{1}}^{2}+3.04{X_{2}}^{2}\\ \;+2.99{X_{3}}^{2}+3.08{X_{4}}^{2} \end{array}$$

**Figure 2:**
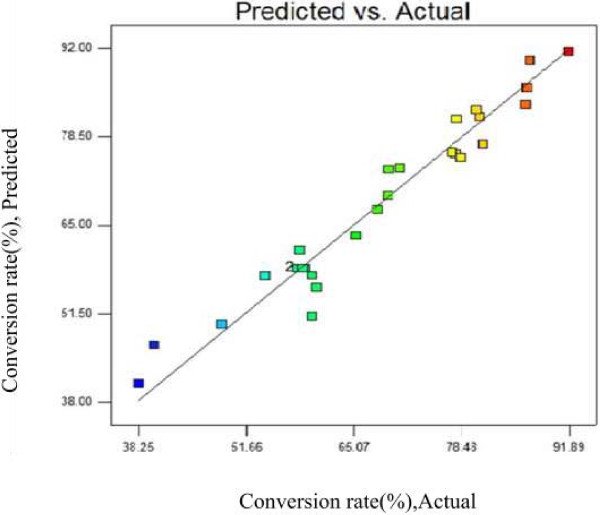
**Plot of predicted and observed conversion rate (%) of D-isoascorbyl palmitate.** (—) Actual; (□) Predicted.

Where *Y* is the response variable (D-isoascorbyl palmitate conversion rate, %), and *X*_1_, *X*_2,_*X*_3,_and *X*_4_ are temperature, molar ratio of D-isoascorbic to palmitic acid, enzyme load and ultrasonic power, respectively. Figure 2 shows the experimental value and predicted conversion rate determined by the model Eq. (1), which indicated that the model was successful in capturing the relationship between actual and predicted responses.

**Figure 3:**
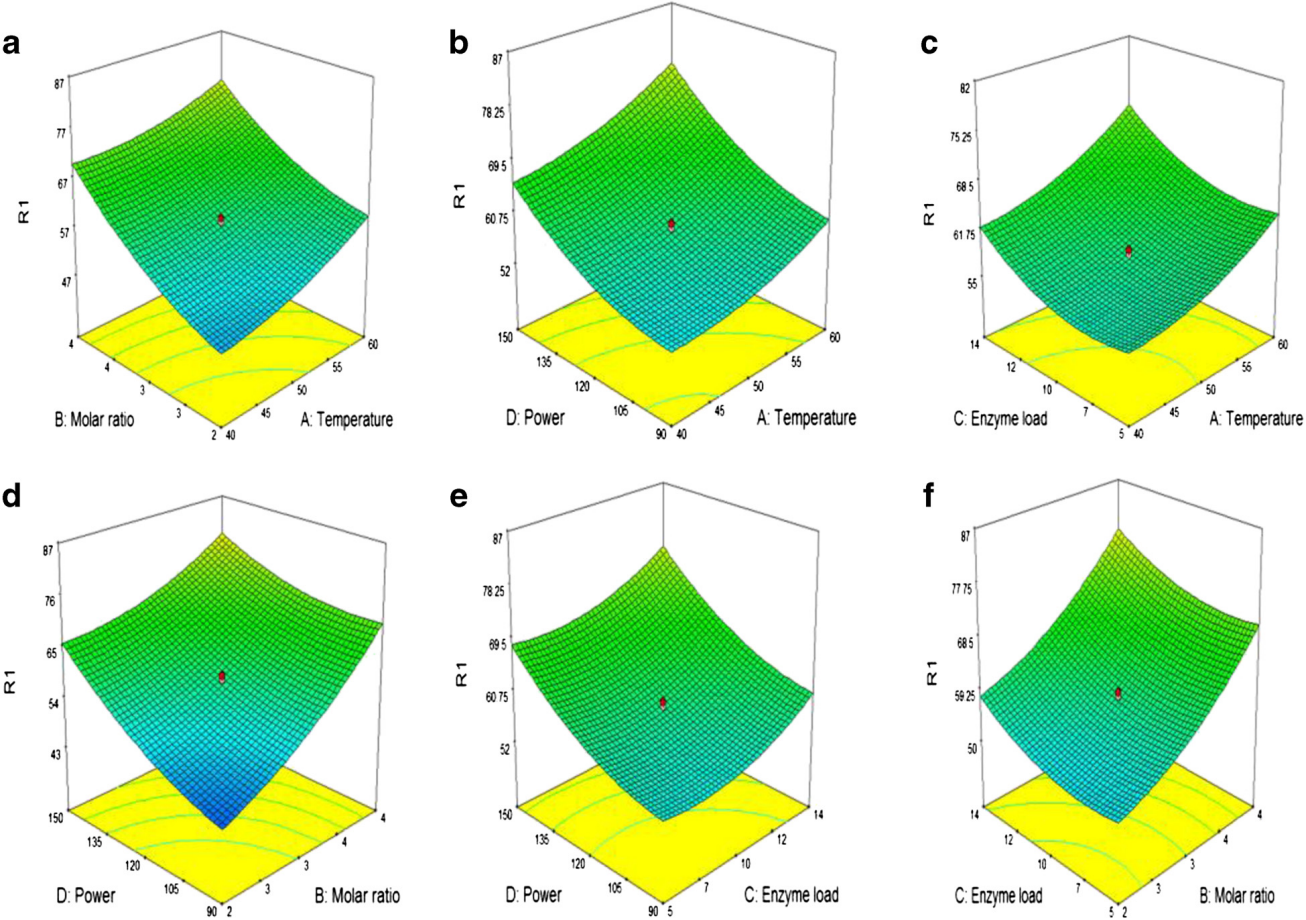
Response surface plots indicating the effect of interaction between reaction parameters on D- isoascorbyl palmitate conversion rate (a) interaction between molar ratio and temperature (b) interaction between temperature and power (c) interaction between enzyme load and temperature (d) interaction between power and molar ratio (e) interaction between enzyme load and power (f) interaction between enzyme load and molar ratio.

3D surface plots of the predicted conversion rate of D-isoascorbyl palmitate were shown in Figure 3. From these figures, the changes of D-isoascorbyl palmitate conversion rate with two variables can be easily obtained by fixing other variables constant at the center values (zero level).

Figure 3a shows the response surface plot on D-isoascorbyl palmitate conversion rate versus temperature and molar ratio of D-isoascorbic to palmitic acid when the ultrasonic power and enzyme load were set at 120 W and 9.5% (zero level), respectively. As shown the 3-D plot, the conversion rate of isoascorbyl palmitate was sensitive to the molar ratio of D-isoascorbic to palmitic acid even with a change from around 38% to 65%. Figure 3b shows that isoascorbyl palmitate conversion rate increased evidently with the increasing of ultrasonic power from 90 W to 180 W. From Figure 3c, an increase in the conversion rate could be obviously achieved with the increase of enzyme load. Our previous study had found that high concentrations of lipase possibly decreased the isoascorbyl palmitate conversion rate due to the agglomeration of enzymes loaded at a higher amount [12]. As for the present study, ultrasound treatment possibly results in the more homogeneous reaction mixture and facilitates dispersion of lipase in substrates reducing the agglomeration [22]. In Figure 3d shows the response surface plot on D-isoascorbyl palmitate yield versus ultrasonic power and molar ratio of D-isoascorbic to palmitic acid when temperature and enzyme load were set at 50°C and 9.5% (zero level), respectively. The conversion rate changes significantly even when molar ratio of D-isoascorbic to palmitic acid and ultrasonic power have the slight increase from 1:1 to 1:4, 90 W to 150 W, respectively. The interaction between enzyme load and ultrasonic power while keeping the other parameters at their center values can be seen in Figure 3e. An increase in ultrasonic power improved the reaction yield for the minimum (0.5%) and maximum (14%) enzyme load. The results were in compliance with those reported by other groups [23-25]. The increase of ultrasonic power within the appropriate range could enhance the enzymatic reaction rate by enhancing the pulsating motions between the enzyme molecules and improving the binding/unbinding interactions with the reactants/substrates. The interactive effect between the enzyme load and molar ratio of D-isoascorbic to palmitic acid can be found in Figure 3f. The conversion rate reached to the maximum level of 77% when the molar ratio was set as 4:1.

Using the Point Prediction function in the Design-Expert 7.1.1 software, the optimal conditions for obtaining the maximum D- isoascorbyl palmitate conversion rate were predicted as: enzyme load 9% (w/w), reaction temperature 61°C, D- isoascorbic-to-palmitic acid molar ratio 1:5 and ultrasound power of 137 W. Under these conditions, the conversion rate reached to the maximum level of 95.09%, which was higher than that of erythorbyl laurate reported by Lee et al. [11] with the conversion rate of 77.81% without ultrasound treatment.

**Figure 4:**
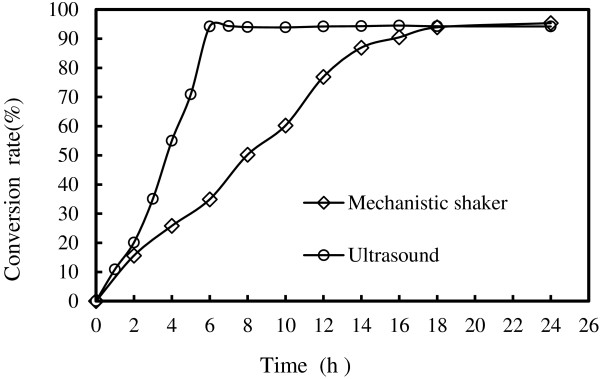
**Comparison between ultrasound and mechanistic shaker on lipase-catalyzed synthesis of D- isoascorbyl palmitate under the optimal conditions.** (The reaction condition of mechanistic shaker was enzyme load of 20% (w/w), reaction temperature of 53°C, D-isoascorbic-to-palmitic acid molar ratio of 1:4, acetone 20 mL, 40 g/L of molecular sieves content, 150 rpm speed. The reaction condition of ultrasound was enzyme load of 9% (w/w), reaction temperature of 61°C, D- isoascorbic-to-palmitic acid molar ratio of 1:5, acetone 20 mL, 50 g/L of molecular sieves content, ultrasound power of 137 W).

To validate this prediction, the above reaction conditions were used for D- isoascorbyl palmitate synthesis for 6 h (Figure 4). The D- isoascorbyl palmitate conversion rate of 94.32 ± 0.17% was finally obtained, which was very close to the predicted value of 95.09%.

As for the D-isoascorbyl palmitate conversion rate, ultrasound treatment slightly increased to 94.32 ± 0.17% compared to that of 94% with the mechanical shaking treatment. However, ultrasound treatment significantly decreased the reaction time from 12 h to 6 h to achieve the comparable conversion rate. The productivity for ultrasound-assisted D-isoascorbyl palmitate synthesis was about 8.67 g L^-1^ h^-1^, which was about 3.96 times of that obtained from mechanical shaking (2.19 g L^-1^ h^-1^).

### Enzyme reuse under the ultrasound-assisted conditions

**Figure 5:**
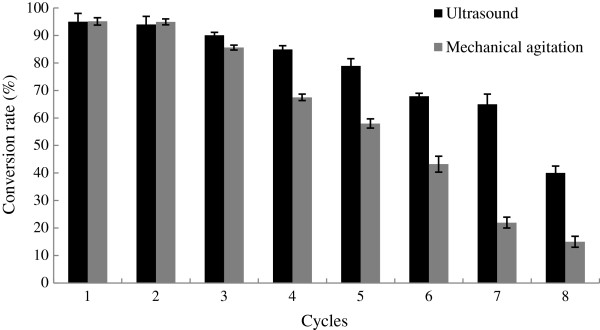
Comparison between ultrasound and mechanistic agitation on reusing of Novozyme 435 for D- isoascorbyl palmitate synthesis.

Enzyme recovery and reuse were still the main challenges during the bio-esterification reactions [26,27]. Therefore, the reuse of the immobilized lipase Novozym 435 was tested. From Figure 5, the conversion rates of D-isoascorbyl palmitate production was 65% and 15.02% after 7-time reuses under the ultrasound-assisted and mechanical shaking treatments, respectively.

### Kinetics of ultrasound-assisted lipase catalyzed D-isoascorbyl palmitate synthesis

**Figure 6:**
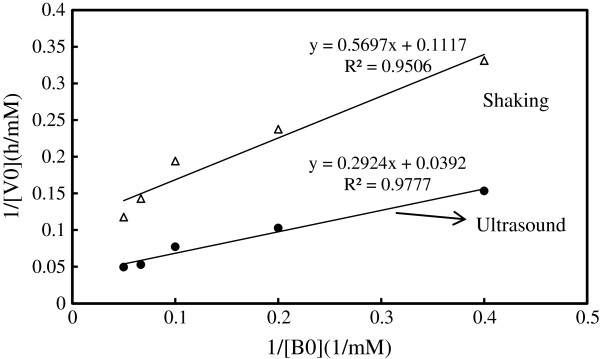
**Lineweaver-Burk plot of reciprocal initial reaction rate against reciprocal palmitatic acid (*B***_***0***_**) concentrations.**

The ping–pong bi–bi kinetic mechanism illustrates alternate binding of substrates and release of products in a bi-substrate reaction with two formed products. It has been the most frequently used for describing the lipases-catalyzed esterification or transesterification process [28,29]. In this experiment, bi-substrate reaction of D-isoascorbic acid and palmitic acid was involved. Michaelis-Menten model still can be used in the present reaction by fixing the D-isoascorbic acid concentration fixed at 2.5 mM [30]. The enzymatic reaction kinetic constant was calculated by changing the palmitic acid concentrations from 2.5 mM to 20 mM. As shown in Figure 6, Lineweaver-Burk plots revealed that the Novozym 435-catalyzed esterification process of D-isoascorbic acid and palmitic acid followed the classical Michaelis-Menten kinetics. The kinetic constants, including the apparent Michaelis constant, *Km* (the Michaelis constant for palmitic acid) and the apparent maximum reaction rate (*v*_max_), were calculated according to equation (2) and the Lineweaver-Burk plot.

(2) $$\frac{1}{v}=\frac{\text{Km}}{v_{\text{max}}}\cdot \frac{1}{\left[S\right]}+\frac{1}{v_{\text{max}}}$$

The curve of initial velocity and palmitic acid concentration shows good linearity. Under the ultrasound-assisted treatment, *Km* and *v*_max_ values were 7.49 mM and 25.64 mM h^-1^, which were are 1.58 times and 2.85 times of those with the mechanical shaking treatment, respectively.

The comparison between ultrasound and mechanical shaking treatments was summarized in Table 3. These treatment methods resulted in the significant differences of reaction parameters, D-isoascorbyl palmitate productivity, and reaction rate. Further investigations will focus on the cost assessments of energy input and development of large scale ultrasound assisted reaction system.

**Table 3:** Comparison of D-isoascorbyl palmitate conversion performance between ultrasound and mechanical shaking conditions

**Items**	**Ultrasound**	**Mechanical shaking**
Reaction time (h)	6	24
Temperature (°C)	61	53
Substrate molar ratio (D-isoascorbic: palmitic acid)	1:5	1:4
Enzyme load (w/w)	9%	20%
Conversion (%)	94.32	95.32
Productivity (g L^-1^ h^-1^)	8.67	2.19
Conversion after enzyme reuse (8 cycles)	40	15.02
Apparent maximum reaction rate (*v*_max_) (mMh^-1^)	25.64	9.01

## Experimental Section

### Materials

D-Isoascorbic acid (purity > 99%) was provided from Parchn Sodium Isovitamin C Co. Ltd (Dexing, Jiangxi, China). Palmitic acid, acetone, hexane and ethyl acetate were of analytical grade and obtained from Sinopharm Chemical Reagent Co., Ltd (Shanghai, China). Lipase of Novozym 435 (EC 3.1.1.3) from *C. antarctica* with the catalytic activity of 10 000 PLU/g (the activity of PLU refers to the millimoles of Lauric acid isopropyl acetate synthesized per minute at 60°C) were purchased from Novozymes (Denmark). This lipase has the optimal reaction temperature of 40-70°C. Methanol was of HPLC-grade purchased from Tedia (Ohio, USA). All reagents were dehydrated by molecular sieve 4 Å (Shanghai world molecular sieve Co., Ltd., Shanghai, China) and filtered using a membrane filter (0.45 μm) prior to use.

### Ultrasound equipment

**Figure 7:**
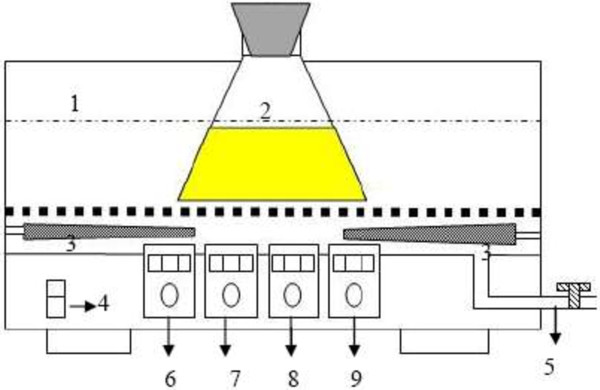
Ultrasound equipment: (1) water-bath; (2) reactor; (3) ultrasonic transducer; (4) power button; (5) water outlet; (6) temperature control panel; (7) ultrasonic timer control panel; (8) water level control panel; (9) ultrasonic output power button.

The reaction was carried out in ultrasound equipment (Model KQ-300DE, Ningbo, China) with 4-Lworking volume (Figure 7). The ultrasound equipment was composed of water-bath, reactor and ultrasonic transducer. The ultrasound power was adjustable from 30 W to 200 W.

### Esterification reaction

The esterification reaction under the mechanical shaking treatment was conducted as described previously [12], the reaction solution included the D-isoascorbic acid (2.5 mM) and palmitic acid with various molar ratios, the immobilized lipase with the concentration from 0.5 to 18.5 (w/w of the substrates amount), 50 g/L of molecular sieve 4 Å. The synthesis reaction was conducted with temperature from 30°C to 70°C in a temperature-controlled shaker at the speed of 150 rpm.

As for the ultrasound treatment, the 150 mL flask was placed within the ultrasonic bath before the reaction was initiated as presented in Figure 7. The reaction solution and compositions are same with those of the mechanical shaking treatment. The reaction temperature was controlled by adjusting the water bath temperature from 20 to 80°C. The ultrasonic power was set from 60 W to 180 W.

All the samples were taken at every 2 h. The sampled reaction mixture was filtered through a membrane filter (0.45 μm), and 20 μL of each aliquot were injected into the HPLC for further analyzing the concentrations of the substrate isoascorbic acid and the produced D-isoascorbyl palmitate.

### HPLC analysis

Determining the concentrations of the produced D-isoascorbyl palmitate, substrate isoascorbic acid was conducted using a Waters Alliance LC-20AT (SHIMADZU, Japan) liquid chromatography connected to a model 2996 (PDA) photo-diode array detector and controlled by LC Driver Ver.2.0 for Waters Empower™ software. The column equipped in the HPLC system was ZORBAX Eclipse XDB-C18 (150 mm × 4.6 mm, 5 μm, Torrance, CA, USA). The mobile phase was methanol/water (90:10, v/v) at 1.0 ml/min flow rate for 30 min. Samples were injected automatically (20.0 μL of each other). The conversion rate (%) was calculated by dividing the initial molar amount of D-isoascorbic acid by the produced molar amount of isoascorbyl palmitate.

### Experimental design

In order to optimize the ultrasound assisted reaction conditions, a 5-level-4-factor Central Composite Design (CCD) including 30 experiments was employed. A Box-Behnken factorial design was used for fitting a second order response surface [31].

Table 4 and Table 1 give the factors, their values, and the experimental design, respectively. A mathematical model was developed to describe the relationships between the response (the D-isoascorbyl palmitate conversion rate) and the varieties (ultrasound assisted reaction conditions) in second order equation. The conversion rate of D- isoascorbyl palmitate was multiply regressed with respect to the ultrasound assisted reaction conditions by using the software Design Expert 7.1.1(Stat-Ease, Minneapolis, MN, USA) as follow:

(3) $$Y=A_{0}+\sum A_{i}X_{i}+\sum A_{\mathrm{ii}}X_{i}^{2}+\sum A_{\mathrm{ij}}X_{i}X_{j}$$

**Table 4:** Process variables and their levels used in CCD

		**Levels**				
**Independent variables**	**Coded symbols**	**-2 -1 0 1 2**				
Temperature (°C)	A(X_1_)	30	40	50	60	70
Molar ratio (D-isoascorbic: palmitic acid)	B(*X*_2_)	1:1	1:2	1:3	1:4	1:5
Enzyme load (w/w)	C(X_3_)	0.5	5	9.5	14	18.5
Ultrasonic power (W)	D(X_4_)	60	90	120	150	180

Where Y is the measured response variable; A_o_, A_i_, A_ii_, A_ij_ are constant regression coefficients of the model, and X_i_, X_j_ (i = 1, 3; j = 1, 3, i≠j) represent the independent variables (the ultrasound assisted reaction conditions) in the form of coded values. The accuracy and general ability of the above polynomial model could be evaluated by the determination coefficient R^2^.

### Enzyme reuse

For evaluating the lipase reuse times, the immobilized lipase was removed by vacuum filtration and the products were recovered for further analysis after finishing each batch reaction. The immobilized lipase was washed twice with 10 mL hexane, and then dried in an oven at 50°C. The collected enzyme was used for the next run of catalyzing the esterification reaction.

### Determination of kinetic constants

To determine the kinetics of the esterification reaction, reaction mixtures were prepared by a D-isoascorbic acid (2.5 mM) with various palmitic acid (2.5-20 mM) in 20 mL of acetone at 50°C. 10% (weight % of substrates) of Novozym 435 was used with 180 W ultrasonic power. Initial reaction rate, expressed as mM produced D-isoascorbyl palmitate per hour, were determined from time course of D-isoascorbyl palmitate concentration by regression analysis of the product concentration and determining the initial slope of the tangent to the curve.

## Conclusions

In this work, ultrasonic assistance of lipase (*Candida antarctica*; Novozym 435) catalyzed synthesis of the D-isoascorbyl palmitate has been studied. Based on the statistical experimental results, the optimized reaction parameters were obtained as follows: enzyme load 9% (w/w), reaction temperature 61°C, D- isoascorbic-to-palmitic acid molar ratio 1:5, and ultrasound power 137 W for 6-h reaction. Under the optimal conditions, ultrasound treatment yielded 94.32% of conversion rate and decreased the reaction time by over 50%. The productivity was about 8.67 g L^-1^ h^-1^, which was 3.96 times higher than that with mechanical shaking system (2.19 g L^-1^ h^-1^). Ultrasound retained the lipase activity well. The catalytic reaction kinetics under an ultrasonic field agreed with Ping-Pong mechanism. The apparent maximum reaction rate (*v*_max_) of the ultrasound treatment was 2.85 times higher than that of the mechanical shaking. Thus, the ultrasound treatment in the D-isoascorbyl palmitate synthesis leads to the improvement of process efficiency.

## Competing interests

The authors declare that they have no competing interests.

## Authors’ contributions

FJ C and WJ S conceived of the study, participated in its design and coordination, and drafted the manuscript. HX Z performed experiments and analyzed results and helped to draft the manuscript. Z W, Q Z, SL Y and Y D performed partial experiments and analyzed results. All authors read and approved the manuscript.
